# Crosstalk between Dendritic Cells and Immune Modulatory Agents against Sepsis

**DOI:** 10.3390/genes11030323

**Published:** 2020-03-18

**Authors:** Guoying Wang, Xianghui Li, Lei Zhang, Abualgasim Elgaili Abdalla, Tieshan Teng, Yanzhang Li

**Affiliations:** 1 Institute of Biomedical Informatics, College of Medicine, Henan University, Kaifeng 475004, China; medwgy@163.com (G.W.); xianghui_li0813@163.com (X.L.); zhlei@henu.edu.cn (L.Z.); 2 Joint National Laboratory for Antibody Drug Engineering, school of Basic Medical Sciences, Henan University, Kaifeng 475004, China; 3 Department of Clinical Laboratory Sciences, College of Applied Medical Sciences, Jouf University, Sakaka 2014, Saudi Arabia; gasimmicro@gmail.com

**Keywords:** dendritic cells, immune modulatory agents, sepsis, innate and adaptive immune, immunosuppression

## Abstract

Dendritic cells (DCs) play a critical role in the immune system which sense pathogens and present their antigens to prime the adaptive immune responses. As the progression of sepsis occurs, DCs are capable of orchestrating the aberrant innate immune response by sustaining the Th1/Th2 responses that are essential for host survival. Hence, an in-depth understanding of the characteristics of DCs would have a beneficial effect in overcoming the obstacle occurring in sepsis. This paper focuses on the role of DCs in the progression of sepsis and we also discuss the reverse sepsis-induced immunosuppression through manipulating the DC function. In addition, we highlight some potent immunotherapies that could be used as a novel strategy in the early treatment of sepsis.

## 1. Introduction

Sepsis is still a life-threatening disease and a major contributor to the public health burden. It is characterized by a systemic inflammatory response syndrome (SIRS) which can lead to organ damage, organ failure, septic shock, and even death. The total burden of sepsis is high with an estimated 19 million clinical cases occurring each year. The annual actual incidence rate of sepsis is apparently far higher and most of these established cases occur in low-income areas. Moreover, due to the increased number of people with advanced age and improvements in the methods of detection, the incidence of sepsis was shown to gradually increase (8–13% per year) in high-income countries. In low-income countries, the mortality rate as a result of septic shock and severe sepsis is almost 60% [1]. Apart from the short-term mortality of patients with sepsis, septic patients undergo plenty of long-term complications which will affect the quality of life and increase the five-year risk of death from subsequent of acute events [2,3,4].

Sepsis is currently considered as a complex deregulation of the inflammatory processes, which lead to compromise the individual’s ability for the containment of infection. The high risk associated with sepsis can be only reduced through interventional therapies [5].

Dendritic cells (DCs) are key components of the innate immune system that are responsible for sensing bacteria or microbial products, processing the antigens, and priming the protective adaptive immune responses through antigen presentation to helper T lymphocytes [6]. The role of DCs has been studied thoroughly in many human diseases, such as autoimmune diseases, while there is limited data on the DCs’ role in human sepsis. During the progression of sepsis, DCs can play a direct role in the aberrant immune response, and increasing the number of DCs or enhancing their functionality may lead to improve the outcome of disease [3,7]. Undoubtedly, understanding the mechanisms of DCs’ response during sepsis will be crucial for developing novel strategies for fighting this disease. Recent studies imply that some novel immunomodulatory agents, including IL-7, IL-15, GM-CSF, IFN-γ, and co-inhibitory molecule blockade can reduce the clinical morbidity associated with severe sepsis and septic shock [8,9,10].

## 2. DC Numbers, Subgroups and Immune Functions in Sepsis

**Figure 1:**
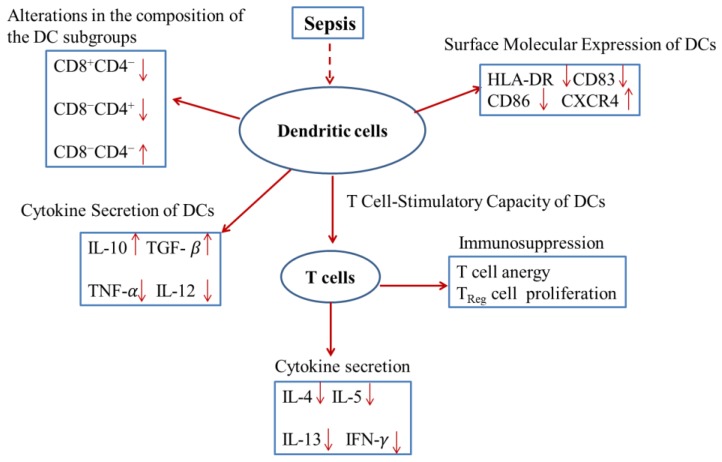
The surface molecules associated with dendritic cell (DC) function are changed during sepsis, and the number of DCs will be decreased, resulting from apoptosis; moreover, the secretion of cytokines in DCs will be changed, which leads to immunological tolerance.

DCs play an important role in the cross-link between innate and adaptive immunity to control microorganism infection. However, they have been shown to contribute to the development of immune suppression during sepsis. The DCs in septic patients undergo multiple alterations as follows: number of cells decreased, alteration of the DC subgroups, as well as alteration of their immune functions (Figure 1).

### 2.1. Sepsis can Lead to Reduction in DC Number

Previous studies of sepsis in animal models and in human patients have shown that the number of DCs was depleted in both lymph and non-lymphoid organs [11]. Using the cecal ligation and puncture (CLP) mouse model of sepsis, the number of CD11c+ DC was found to be lower in the spleen and lymph nodes than that in sham operation groups [12]. In peripheral organs of septic patients, clinical trials have shown that the number of interstitial DCs was remarkably lower than that in non-septic patients [13]. Moreover, it was demonstrated that the low DC count during the early stage of sepsis is closely related to the severity of sepsis. Therefore, the number of DCs can be a useful prognostic maker to evaluate the severity of disease and host response status against infection [14,15]. Sepsis can induce a numerical loss of DCs without significantly changing their maturation status, suggesting that the reduction of DC number may be mediated by an apoptosis process.

### 2.2. Alterations in the Composition of the DC Subgroups during Sepsis

DCs comprise two major distinct subsets: plasmacytoid DCs (pDCs) and myeloid DCs (mDCs), which are also called conventional or classical DCs (cDCs) [16]. The cDCs are professional antigen presenting cells characterized by an incredible ability to capture antigens and the expression of high levels of major histocompatibility (MHC) class II and co-stimulatory molecules which enables them to stimulate T cells [9]. 

Based on the surface expression of CD4 and CD8, DCs can be divided into three subgroups including CD4^–^CD8^–^, CD4^+^CD8^–^, and CD4^–^CD8^+^ [17]. Mice treated with CLP or sham operation for 36 h were used to count the number of the three major subpopulations of DCs. The control mice treated with sham operation exhibited the proportion of three DC subpopulations as follows: subpopulation CD4^+^CD8^–^ (72%), CD4^–^CD8^+^ (11%), and CD4^–^CD8^–^ (15%) [18]. In contrast, CLP-treated mice displayed a significant decrease in the number of CD4^+^CD8^–^DC and CD4^–^CD8^+^ DCs when compared with control groups. Meanwhile, in the spleen of both CLP and sham operation groups, the number of CD4^–^CD8^–^ DCs remains unchanged. Therefore, the disappearance of splenic DCs during sepsis can account for a profound reduction of two special DC subgroups.

### 2.3. Sepsis can Lead to Functional Impairments in DCs

Sepsis is not only the cause of the decreased DC count, but it can also lead to functional limitation. These cells can lose their ability to produce inflammatory cytokines upon stimulation [19]. It was demonstrated that CD11c^+^ DCs harvested from the CLP model are impaired to expressed IL-12p40 and tumor necrosis factor-alpha (TNF-α) upon stimulation with lipopolysaccharide (LPS) or CpG, implying that sepsis has the ability to change DCs’ response to a Toll-like Receptor (TLR) agonist [20,21]. Similarly, splenic DCs from septic mice are unable to secrete IL-12, but they can release a significant amount of IL-10 compared with DCs obtained from control mice, suggesting that sepsis can abrogate DCs inducing Th1 cell polymerization [22]. However, DCs restore the capacity to promote the T cell proliferation during sepsis [23,24]. It was found that immature DCs from patients with sepsis and health donors have a similar ability to induce T cell proliferation, but the mature DCs did not [25]. Lastly, the expression levels of the B and T lymphocyte attenuator (BTLA), a co-inhibitory receptor, was found to be improved in immature and mature DCs in the peritoneum after CLP, which can result in an increased bacterial burden and severity of sepsis [26,27]. Therefore, BTLA may be considered as a potential therapeutic target, as it was shown that antibody blockade of BTLA can protect mice from LPS-induced septic shock [28,29].

## 3. Immunization of Sepsis with DC as the Target

### 3.1. Increasing the Number of DCs in Vivo 

The reduction of DCs’ number is the dominant cause of immunosuppression and opportunistic infections during sepsis, which is closely related to an unsatisfactory prognosis. The lack of differentiation of peripheral blood mononuclear cells into DCs during sepsis can lead to the destruction of the dynamic balance between innate and acquired immunity. Therefore, increasing the number of DCs has potential as an immunoregulatory treatment for sepsis.

Fms-related tyrosine kinase 3 ligand (Flt3L) is a hemopoietic cytokine that serves as a DC growth factor, and can enhance the regeneration of DCs [30]. Flt3L receptor, named Flt3R, has also been shown to stimulate the expansion of progenitor cells and differentiation of both myeloid and lymphoid cells into DCs. It was also found that Flt3L treatment can augment DCs to release multiple cytokines, such as IFN-γ and IL-12 secretion, and to stimulate robust Th-1 responses [31,32]. In vivo studies demonstrated that the administration of Flt3L can greatly increase the DCs’ number and promote mice resistance to burn wound infection with *P. aeruginosa* [33,34]. In addition, Flt3L treatment can also prevent the decline of splenic CD4^+^ and CD8^+^ T cells, which has significantly improved the survival rate of the septic mice [35,36]. These results suggested that amplification and functional enhancement of DC in vivo by Flt3L treatments can result in the enhancement of protective immune responses.

Complement protein C5a is a key component of proinflammatory mediators, which can induce IL-12^+^ DC migration from the peritoneal cavity to the peripheral blood and lymph nodes [37,38]. During sepsis, it was observed that the expression of C5a was excessively increased, which has harmful effects to the host [39]. Blockade of C5a can play a protective role against sepsis through increasing the amount of IL-12^+^ DCs in the peritoneal cavity [40]. 

### 3.2. Anti-DC Apoptosis

DC apoptosis plays a key role in the homeostasis process of the immune system during sepsis. Imbalance reducing in the number of DCs will decrease T lymphocyte proliferation and trigger the emergence of an immunosuppressive state [41,42]. Therefore, inhibition of DC apoptosis would improve the immune function and enhance the survival rate of septic patients. Some of the immune regulatory molecules such as cytokines, microRNAs (miRs), B cell lymphoma 2 (BCL-2), CD40 ligand (CD40L), TNF-related activation-induced cytokine (TRANCE), and histamine have been shown to negatively regulate DC apoptosis.

BCL-2 is an anti-apoptotic factor and key regulator of DC lifespan and immunogenicity. Bcl-2 transgenic mice exhibited limited number of DC apoptosis and improved their survivability during severe sepsis in comparison with wild type mice [43]. Overexpression of BCL-2 can increase DC survival and maintain the T cell activation and differentiation into the Th1 cell phenotype [44]. 

MiRs are short non-coding RNAs that regulate multiple biological processes via post-transcriptional regulation of gene expression. MiR-146a and miR-146b expression were found upregulated upon human monocyte differentiation into DCs. Silencing of miR-146a and/or miR-146b in immature DCs and mature DCs can dramatically block DC apoptosis and enhance proinflammatory cytokine production such as IL-12p70, IL-6, TNF-α, and IFN-γ [45]. 

CD40L is a transmembrane glycoprotein that is found on the surface of CD4+ T cells, which is crucial for CD4+ T cell activation when it binds with CD40 on the DC. It has been demonstrated to downregulate the expression of Bcl-2 in DCs and inhibit Fas-mediated apoptosis [46,47,48]. 

TRANCE, an apoptosis-inducing ligand of the TNF family, can augment the expression of Bcl-xL, leading to inhibition of murine DC apoptosis [49,50]. Inhibition of DC apoptosis by TRANCE can improve the immune function of the body and inhibit sepsis sequelae. 

Histamine is a proinflammatory mediator that is found in performed state within the granules of basophils and mast cells. It was shown that histamine can abolish DC apoptosis by inhibition of caspase-3 cleavage through a mechanism dependent on protein kinase C activation [51]

### 3.3. Function Modification of DCs

DCs can secrete cytokines and manipulate lymphocyte function, which is an important manifestation of sepsis-associated immunosuppression (Figure 1). Therefore, the regulation and modification of DC impaired function would improve the immune function of sepsis. Some molecules have been shown to be hopeful targets for improving the function of DCs and prolonging their life during sepsis progression. These molecules include high mobility group protein 1(HMGB1), CD155, toll-like receptor 4 (TLR4) and TLR2. In addition, phospholipase A2, miR-142-3p, SHARPIN, and glucocorticoids also possess the ability to correct impaired function of DCs (Table 1).

HMGB1 derived from DCs is a major regulator of late and sustained cytokine storm [52,53]. Administration of HMGB1 to mice can cause lethal organ damage similar to that seen in sepsis. Therefore, HMGB1 is a decisive inflammatory mediator and can be considered as a new target in the treatment of sepsis. Neutralization of HMG1 with an antibody results in reduced mice succumbs during sepsis. Likewise, ablation of HMGB1 secretion in human DCs via siRNA, which targets the short acetylcholine receptor (ACHR) binding peptide, leads to decreased human cytokine storm and prevention of human lymphocyte apoptosis. Furthermore, silencing of HMGB1 expression can suppress CLP-induced humanized mice death [54].

The expression of CD155 on DCs was significantly boosted in septic mice. Administration of anti-CD155 antibody can reverse DC dysfunction and reduce morbidity of mouse sepsis models. Mechanistically, blockade of CD155 can efficiently increase the expression of pro-inflammatory molecules, such as TNF-α and IL-6, but it decreased the levels of anti-inflammatory IL-10. Nevertheless, the overexpression of CD155 can significantly increase the production of IL-10 and inhibit the production of IL-12P40 and IL-12P70, suggesting that the expression of CD155 on dendritic cells can promote immunosuppression by regulating the production of cytokines in sepsis [55].

TLR4, TLR2, and TLR9 play a central role in the response to intra-abdominal sepsis. TLR4 and TLR2 are involved in the control of splenic DC apoptosis during polymicrobial sepsis, suggesting that modulation of DC-specific TLR4/TLR2 signaling may be a new therapeutic strategy for the treatment of sepsis [56]. One of the TLR4 antagonists, named FP7, can inhibit LPS-induced cytokine production and DC maturation. In addition, blockade of TLR4 signaling can protect the mice from lethal viral sepsis [57,58]. However, activation of TLR2 signaling is crucial for improving the immune function during sepsis. Consistently, two TLR2 agonists, named MALP-2 and Pam3Cys, provoke the cytokine and chemokine secretion and prevent the sepsis-induced early depletion of splenic DC [59]. TLR2-derived peptides can also enhance antigen-induced DC maturation, IL-12 production, and differentially affect DC cytokine profile upon antigen stimulation [60,61]. Some studies reported that TLR9 is essential for the uptake and intracellular killing of the bacteria during infection with *K. pneumonia*. In addition, TLR9 can play a role in recruiting and activating DCs, which is required for the optimal activation of bactericidal activity [62].

Secretory phospholipase A2 (spla2) is an indispensable enzyme that catalyzes the production of lysophospholipids and free fatty acids by hydrolyzing phospholipids at the sn-2 site [63]. Administration of sPLA2 on DCs can result on activation of AP-1, NFAT, and NF-κB, which control the expression of multiple genes involved in immune regulation. Importantly, sPLA2 can bind to specific membrane receptors on DCs to mobilize lipid mediators and induce DC maturation [64]. 

MicroRNA can also be a potential target for the immunoregulation of DCs. For example, miR-142-3p was found to decrease LPS-induced death through targeting and inhibiting IL-6 expression [65]. 

SHANK-associated RH domain-interacting protein (SHARPIN) is an endogenous inhibitor of caspase-1 activation, and the interaction of SHARPIN–caspase-1 can lead to suppress the release of mature cytokines [66]. SHARPIN can also positively regulate NF-κB signaling, cytokine production, as well as the induction of Th1 differentiation by DCs [67,68]. 

Finally, glucocorticoids are an important member of steroid hormones which regulate various essential metabolic, cardiovascular, as well as homeostatic functions [69]. Mice with knockdown glucocorticoid receptor in DCs exhibited high susceptibility to endotoxin-induced septic shock and death. Endogenous glucocorticoids can blunt LPS-induced inflammation and promote tolerance by suppressing DC IL-12 production [70].

## 4. New Approaches: Immunotherapies in Sepsis

Sepsis is one of the main factors leading to death of critically patients in intensive care units (ICUs), and the hospitalization rate for sepsis have constantly increased, even though advances in supportive care mortality are still high. Therapy approaches developed against the pro-inflammatory stage have failed to show clinical efficacy [77,78,79,80,81]. Hence, new treatment strategies for sepsis targeting host immune response are urgently needed. The profound immunosuppression is one key factor in the sepsis pathophysiology, which often engenders secondary fungal, bacterial, or viral infections [82,83,84,85]. The following contents of this review will highlight the immunomodulators that improve T cell immune responses during sepsis, such as IL-7, PD1/ PDL1-specific antibodies, IFN γ, G-CSF /GM-CSF, IL-15, IL-1ra, and anti-IL-6 antibody (Table 2).

### 4.1. Recombinant Human IL-7

IL-7, a pluripotent cytokine, is known as a master of the immune system because of its special role in immunotherapy [86]. IL-7 has a potent anti-apoptotic activity, which can abolish sepsis-induced apoptotic depletion of CD4+ and CD8+ T cells [87]. In a murine model of sepsis, IL-7 treatment resulted in the upregulation of the anti-apoptotic protein Bcl-2 and downregulation of pro-apoptotic proteins such as Bim and Puma [88]. IL-7 is also capable to prevent the sepsis-inducing depression of T-cell factors such as IFN-γ and enhance the expression of cell adhesion molecules, such as very late antigen (VLA)-4 and lymphocyte function-related antigen (LFA)-1 [89,90]. 

### 4.2. PD1/ PDL1-Specific Antibodies

Programmed death-1 (PD-1), a co-inhibitory receptor with similarities to BTLA and T-lymphocyte antigen (CTLA)-4, exerts an inhibitory function by regulating the balance among T cell activation, tolerance, and immunopathology, which is often connected with the phenomenon of “T cell exhaustion” [91,92]. Blockade of PD-1 or its ligand PD-L1 hold great potential in reversing immunosuppression in sepsis via arresting lymphocyte apoptosis and preventing monocyte dysfunction [93,94]. Moreover, blockade of PD1–PDL1 or deficiency can significantly elevated the survival rate and reduced the mortality rate in animal models with sepsis caused by *C. albicans* [95]. It was noted that the decreased levels of MHC II expression in DC in the CLP model can be reversed by both anti-PD-1 and anti-PD-L1 treatments [96]. Importantly, PD-1/PD-L1 can be used as a candidate biomarker for monitoring the treatment of septic patients, as well as highly potential targets to maintain the normal function of adaptive immunity [97]. 

### 4.3. IFN- γ

IFN-γ is a decisive proinflammatory cytokine responsible for the activation of macrophages and monocytes, which play a key role in bacterial elimination during sepsis. Clinical studies demonstrated that the production of IFN-γ by T cells was decreased significantly during sepsis [98]. Moreover, treatment with recombinant IFN-γ has been shown to reverse monocyte dysfunction and rescue the septic patients [99,100]. Although IFN-γ, as a potential immunotherapeutic agent, provides a real improvement for sepsis patients, it may be dangerous when administered in the pro-inflammatory phase of sepsis by exaggerating the stimulation of monocytes and forming a vicious inflammation cycle known as hyper-inflammation [101]. Thus, IFN-γ therapy can achieve better results with a time-dependent strategy and in combination with granulocyte–monocyte colony-stimulating factor (GM-CSF), granulocyte colony-stimulating factor (G-CSF), or IL-7 and/or IL-15, which have the ability to enhance immune response, diminish secondary infections as well as improve long-term survival as sepsis recovery evolves [102].

### 4.4. G-CSF and GM-CSF

There are a few drawbacks in neutrophil function that occur in patients with sepsis resulting from hospital-acquired and community-acquired pneumonia. However, G-CSF has been shown to increase the number of neutrophils and modify their activity and function for better pathogen killing capacity, which will be instrumental in patients with neutropenia-related sepsis [103]. Additionally, G-CSF can also play a critical role in enhancing the activity of other immune cell functions, such as monocyte and macrophage. Meanwhile, a previous study demonstrated that intravenous administration of G-CSF was associated with long duration of survival rate in severe septic patients [104]. 

GM-CSF is a 23-kD heterodimer cytokine which can stimulate the stem cells to differentiate into neutrophils, monocytes, and macrophages [105]. Additionally, it has been shown that GM-CSF can modulate DC differentiation to reach the state of tolerance, which can boost regulatory T-cell number and function [106]. Recombinant GM-CSF therapy in immunosuppressed patients with sepsis can restore the HLA-DR expression and TNF production [107]. A meta-analysis study demonstrated that GM-CSF can reduce the rate of infection [108]. Furthermore, it was reported that at least two clinical trials of GM-CSF for the treatment of sepsis are currently enrolling patients (NCT01374711 and NCT01653665) [109].

### 4.5. IL-15

IL-15 is a pleiotropic cytokine that is closely correlated with IL-7. IL-15 plays a key role in the regulation of effector and memory T cells, natural killer (NK) cells, as well as natural killer T (NKT) cells. These properties make IL-15 an attractive candidate for immunotherapy in sepsis [110]. In mouse models of sepsis, administration of IL-15 can significantly inhibit sepsis-induced apoptosis of immune competent cells through boosting Bcl-2 expression [111]. However, it was shown that IL-15 has potential toxicity effects through causing liver injury and cachexia [112]. Therefore, IL-15 can be used in combination with other immunotherapeutic agents for sepsis treatment, such as antibodies that block PD-1 or CTLA-4, which have been found to diminish IL-10 production and PD-1 expression on CD8+ T cell [113].

### 4.6. IL-1ra

The IL-1 receptor antagonist (IL-1ra) is a competitive inhibitor that prevents IL-1 (including IL-1α and IL-1β) from interacting with the IL-1 receptor1 (IL-1R1) [114,115]. Silencing of IL-1ra can lead to numerous rampant inflammatory diseases including sepsis and Muckle–Wells syndrome. In septic mice, administration of IL-1ra could prevent IL-1β-induced septic shock [116]. Moreover, mice deficient in IL-1ra were more susceptible to endotoxemic death, while administration of recombinant human IL-1ra (rhIL-1ra) can significantly improve the survival of septic mice, providing evidence that IL-1 might be a promising therapeutic target against sepsis [117]. 

### 4.7. IL-6

IL-6 was determined to be implicated in the induction of thrombosis, vascular leakage, and multiple organ dysfunctions during severe sepsis [118]. In septic mice, IL-6 gene knockout can significantly ameliorate pulmonary function, edema formation, and lung pathologies. The administration of anti-IL-6 antibody within 4 h after CLP was found to improve survival in a murine sepsis model. Moreover, the serum level of IL-6 is <4 pg/mL in healthy individuals, but it increases to >1000 pg/mL in severe sepsis, which indicated that IL-6 can serve as an excellent biomarker of severity and prognostic indicator of outcome for septic patients [119].

## 5. Conclusions

DCs are defined as professional antigen-presenting cells of the immune network, and play a key proinflammatory role during sepsis. Strategies focused on inhibiting the dysfunction of DCs could increase survival in sepsis. Administration of DCs can efficiently arrest the induction of the immune suppression function of the residual DCs in septic mice, it diminished the proliferation and differentiation of Treg cells and suppressor T cells via a combination of factors like indoleamine 2,3 deoxygenase (IDO) and IL-10, enhanced the immune clearance of sepsis-causing pathogens, and eventually reduced organ damage, thereby improving the therapy effect [130,131]. A few immunotherapies that aimed to enhance DCs’ function have been shown to be capable of mitigating the disease symptoms. The effect of DC differentiation, maturation, migration, as well as information transfer through other cytokines during sepsis has yet to be further explored. Furthermore, the dose and safety aspects of DC treatment need further studies. Some recent reports also provide remarkable insights into novel strategies of immunotherapies contributing to alleviate the clinical morbidity associated with sepsis and septic shock.

## Figures and Tables

**Table 1:** Immunization of sepsis with DCs as the target.

Immunotherapy Agents	Major Functions	Ref.
FLT3L	Increasing the numbers of DCs	[31,33,34,36,71,72]
BCL-2	Inhibiting Fas-mediated DC apoptosis	[73]
CD40L	Inhibiting Fas-mediated DC apoptosis	[47,48]
TRANCE	Inhibiting Fas-mediated DC apoptosis	[50]
Histamine	Inhibiting DC apoptosis	[51]
Anti-HMGB1 antibody	Reducing cytokine storm	[52,53,54]
Anti-CD155 antibody	Reverse DC dysfunction	[55]
Anti-C5a antibody	Improving survival of sepsis	[37]
TLR2-derived peptide	Promoting DC maturation	[74]
sPLA2	Increasing the IFN-*γ*secretion	[63,64,75]
miR-142-3p	Promoting the expression of IL-6 and then reducing endotoxin-mediated mortality	[65]
SHARPIN	Induction of Th1 differentiation by DCs	[67,68]
TLR4 agonist	Inhibiting LPS-induced cytokine production	[57,59]
Glucocorticoids	Reducing IL-12 production of DCs	[69,70]
CYT387	Inhibiting LPS-induced cytokine production	[76]

Ref.—Reference; FLT3L—Fms-related tyrosine kinase 3 ligand; BCL-2—B cell lymphoma 2; CD40L—CD40 ligand; TRANCE—TNF-related activation-induced cytokine; HMGB1—high mobility group protein 1; TLR2—toll-like receptor 2; TLR4—toll-like receptor 4; SHARPIN—SHANK-associated RH domain-interacting protein.

**Table 2:** New approaches: Immunotherapies in sepsis.

Immunotherapy	Major Functions	Ref.
GM-CSF	Improving the production and function of neutrophils and monocytes.	[120,121]
IL-7	Inducing the proliferation of naive and memory T cells; decreasing Sepsis-induced lymphocyte apoptosis and reversing sepsis-induced depression of interferon γ	[89,122,123]
IL-15	Increasing NK cell, T cell, NKT cell proliferation and activation	[111,112]
IFN-γ	Reversing monocyte dysfunction; Increasing the numbers of IL-17-expressing CD4^+^ T cells	[99,124,125]
IgGAM	Improving pathogen recognition and anti-apoptotic effects	[126]
Mesenchymalstem cells	Augmenting bacterial clearance	[127,128]
PD1/PDL1-specific antibodies	Improving IFN-γ production and decreasing apoptosis of T cells.	[96]
IL-1ra	Preventing IL-1β-induced septic shock	[115]
anti-IL-6 antibody	Improving survival in sepsis model.	[129]
